# What affects rural households’ entrepreneurial performance: Formal or informal finance availability?

**DOI:** 10.1371/journal.pone.0343040

**Published:** 2026-02-13

**Authors:** Zhilue Xiang, Qiaoyue Yin, Xizhi Xue, Wanting Zhang, Xia Wang

**Affiliations:** 1 School of Economics and Management, Nanjing University of Industry Technology, Nanjing, China; 2 School of Business, Nanjing Normal University, Nanjing, China; 3 School of Finance, Nanjing University of Finance and Economics, Nanjing, China; 4 School of Computing and Software, Nanjing University of Industry Technology, Nanjing, China; 5 School of Economics, Fuyang Normal University, Fuyang, China; University of Professional Studies, GHANA

## Abstract

This study explores the role of financial availability in the growth and development of rural household entrepreneurship, particularly beyond the start-up phase. Focusing on entrepreneurial rural households, the study uses CFPS data to determine how financial availability influences their performance. Results reveal that formal finance significantly contributes to the growth of entrepreneurial performance, whereas informal finance does not. Further, the positive impact of formal finance exhibits notable regional heterogeneity. These findings suggest policies should strengthen formal finance, adopt regionally differentiated support strategies, and guide the complementary development of informal finance to foster sustainable rural entrepreneurship growth.

## 1. Introduction

Entrepreneurship in the global context is of great significance for economic and social development [1]. In rural areas, households’ entrepreneurial activities can effectively promote the progress of the rural economy, achieve inclusive economic growth, reduce poverty, and narrow the income gap between urban and rural areas [2–5]. In this regard, governments have introduced policies to provide financial, technological, and market support for entrepreneurship among rural households, with the aim of guiding and encouraging them to engage in innovative agriculture, rural tourism, handicraft production, and renewable energy use. However, entrepreneurship in emerging economies faces more serious challenges compared with developed economies [6]. Due to limited resources, asymmetric market information, inadequate infrastructure, and lack of entrepreneurial skills, it is often difficult for rural households to cross the threshold of ‘new entry deficiencies’, resulting in a high rate of premature entrepreneurial projects [7]. At the same time, the entrepreneurial activities of many rural households still remain at the ‘survival’ or ‘self-employment’ level, lacking long-term planning and strategic vision, with relatively low entrepreneurial level and performance, making it difficult to achieve significant income-generating effects [8,9]. Therefore, how to help rural households’ entrepreneurship develop sustainably, improve entrepreneurial performance, and realize income generation has become an important issue that needs to be solved urgently.

Finance plays a vital role in entrepreneurial activities. Liquidity constraints may hinder potential entrepreneurs, making it difficult for them to start or expand their entrepreneurial ventures [10]. Entrepreneurs’ capital mainly comes from internal accumulation and external finance; when internal funds are insufficient, external finance becomes decisive for project implementation and development [11]. This finding is particularly relevant for rural households, who may lack the substantial capital required to initiate or scale up their businesses [12].

On the one hand, illiquidity and finance constraints pose significant challenges to rural households seeking to start or expand their entrepreneurial ventures. Research has consistently shown that these financial barriers can impede not only the initial startup of a business but also its subsequent development and performance. Credit constraints severely affect the financial mobility of rural households with entrepreneurial intentions, as the wealthy households have more capital to realize their entrepreneurial projects than the poor households [13,14]. Credit constraints significantly decrease the probabilities of households starting businesses [15]. This suggests that limited access to credit can severely impede rural households’ ability to initiate and sustain entrepreneurial ventures. Financial constraints play an important role in shaping the patterns of entrepreneurship in Thailand, where wealthier households are more likely to start and invest in businesses due to their greater access to credit [16]. Furthermore, policy-led bank branch withdrawal in rural China has a negative impact on credit availability, which impedes self-employment choices among households [17]. These studies indicate that credit constraints not only affect the initial stages of entrepreneurship but also persistently hinder entrepreneurial development and performance [18].

On the other hand, strong financial support and a favorable financial environment have been shown to facilitate entrepreneurship among rural households, leading to improved entrepreneurial performance. Financing is fundamental for entrepreneurs at every stage of their business lifecycle, from initial start-up to subsequent growth and expansion [19,20]. Entrepreneurs need capital to fund their business ideas, cover operational costs, and invest in resources that can help them grow and scale their ventures. Finance not only provides the means to turn business ideas into reality but also serves as a buffer against potential risks and uncertainties that are inherent in starting a new business [21]. Several studies have emphasized the positive relationship between financial availability (FA) and entrepreneurial performance. When the entrepreneur’s own funds are insufficient, adequate availability of finance can alleviate productive financial constraints and is an important determinant of rural household entrepreneurship [22]. The mechanism of the role of finance availability on entrepreneurship lies in the provision of liquidity support. Based on survey data and empirical facts from both developing and developed countries, the access to different kinds of credit products can be helpful to the entrepreneurship of rural households, such as microcredit for disadvantaged groups or even non-business consumer credit [23,24]. As digital technology advances, the role of digital finance in supporting entrepreneurship is increasingly garnering scholarly attention [4,25–27].

Findings of the above two sides heighten the key role of improving financial availability for the development of rural households’ entrepreneurship. Despite the importance, financial resources are not readily available in rural areas over the developing countries [28]. Rural areas often lack the financial infrastructure and services that are available in urban areas [12]. Meanwhile, rural households, as a vulnerable population, are particularly susceptible to credit discrimination due to their low income levels, limited collateral, and high perceived risk [29,30]. It is noteworthy that rural financial markets in developing countries operate under a dual-track system, whereby entrepreneurial farmers can access capital through formal channels (such as rural commercial banks) or through informal channels (such as relatives or local moneylenders) [31]. However, the literature on the differential effects of these two channels remains contentious, with divergent conclusions stemming from contextual variations and differing research perspectives:

A part of studies argue that informal finance is more useful than formal finance for rural households’ entrepreneurship. Based on the endogenous nature of informal finance within rural social trust [32], these studies reveal that informal finance better aligns with the risk characteristics of rural household entrepreneurial activities and is more readily accessible, thereby meeting the financing needs of rural entrepreneurial households [33–36]. On the contrary, another part of the studies support the greater dominance of formal finance over informal finance. Given the formal financial sector’s strengths in robust supply capacity and low interest rates, these studies contend that formal financing plays a greater role in promoting rural household entrepreneurship [17,37,38].

The contradictory finding in the literature stems fundamentally from two significant limitations. On the one hand, most studies fail to clearly distinguish between different stages of entrepreneurship [39], conflating the impact of formal versus informal finance on early-stage entry with its effect on later-stage performance growth. It is noteworthy that the mechanisms through which finance influences entrepreneurial entry fundamentally differ from those affecting later-stage performance, owing to the unique objectives and constraints of each phase [40]. During entry, the core challenge lies in overcoming initial capital shortages and credit exclusion, enabling rural households to cross the ‘0 to 1’ threshold [20]. Post-entry, however, requires ‘funding fit’ (whether capital aligns with expansion needs) and dynamic capability building to achieve ‘1 to N’ development [41,42]. On the other hand, existing research predominantly confines the mechanism through which financial availability influences rural households’ entrepreneurship to alleviating capital constraints [43], overlooking other potential non-capital mechanisms such as impacts on entrepreneurial intent, motivation, and risk attitudes. Rural households’ entrepreneurial activities and developmental performance are shaped by multiple factors [44–48]; neglecting these potential non-capital mechanisms limits the literature’s comprehensive understanding of finance’s role.

This paper attempts to provide useful answers to the above research controversies and to remedy the shortcomings of the existing studies. We focus on rural households at the stage of entrepreneurial development, to explain the logical mechanism of the impact of financial availability on the growth of entrepreneurial performance of rural households from the aspects of behavioral willingness and behavioral conditions. We compare and analyze the difference in the impact of formal financial availability (FFA) and informal financial availability (IFA), and carry out empirical tests using the CFPS survey data. This study seeks to make several marginal contributions. Firstly, it endeavors to identify the distinctions in the effects of formal versus informal financial availability on entrepreneurial performance. Secondly, by extending the analysis to the post-startup phase, it contributes to a more holistic understanding of the entrepreneurial journey of rural households. Lastly, by exploring additional mechanisms beyond financial constraint alleviation, it offers insights into how financial availability shapes entrepreneurial intentions and behaviors over time. The subsequent structure of this paper is organized as follows: firstly, the mechanism analysis is carried out and the research hypothesis is formulated. Secondly, the data sources, variable selection and descriptive statistics of the empirical study are introduced. In addition, empirical tests and analyses are conducted using econometric models. Finally, research conclusions are obtained and corresponding policy recommendations are made.

## 2. Theoretical model analysis and research hypotheses

**Fig 1:**
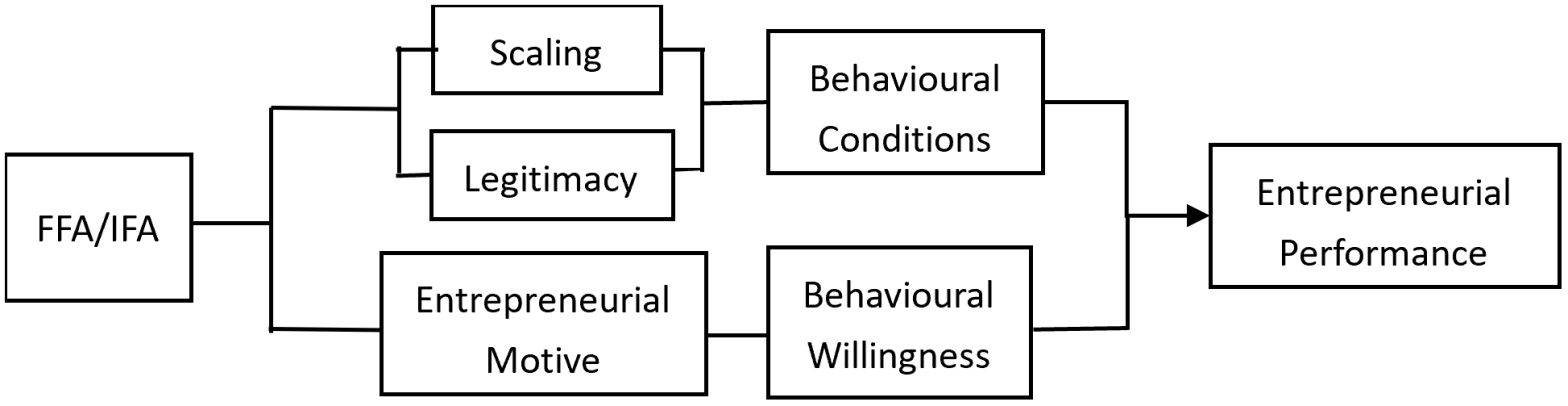
The logic of the role of financial availability.

The Theory of Planned Behavior suggests that individual behavior is not only influenced by behavioral intentions, but also by the opportunity to perform the behavior as well as the actual control conditions such as resources [49]. Accordingly, the enhancement of rural household entrepreneurial performance needs to simultaneously satisfy the following conditions: firstly, entrepreneurial rural households have sufficient willingness to enhance their performance, and secondly, they satisfy certain conditions to enhance their entrepreneurial performance. As illustrated in Fig 1, the logical mechanism of credit availability affecting the improvement of entrepreneurial performance of rural households can be analyzed from the two aspects of behavioral willingness and behavioral conditions.

### 2.1. Theoretical analysis of behavioral willingness

For entrepreneurial rural households, financial availability in the form of loans or investments serves as a critical resource that can significantly enhance their entrepreneurial performance. The obligation to repay these funds within a specified timeframe creates a subjective incentive for rural households to increase their earnings through entrepreneurship. This repayment pressure translates into a strong entrepreneurial motive, driving rural households to actively search for opportunities, monitor relevant business information, evaluate the potential value of these opportunities, and efficiently allocate resources to capitalize on them [50,51]. Throughout the entrepreneurial journey, rural households often encounter numerous challenges and obstacles, testing their resilience and determination. The entrepreneurial motive fostered by financial obligation acts as a continuous source of motivation, sustaining their passion and confidence even in the face of adversity [52]. This motive aligns with the rural household’s overarching goal of successfully repaying the loan and achieving financial stability, guiding their decision-making processes, opportunity recognition, creativity, and overall business management [53]. The entrepreneurial motive not only shapes the rural household’s initial business concept and model but also influences their strategic direction and adaptive capabilities as the business evolves. By maintaining a clear and compelling entrepreneurial goal, rural households are less likely to deviate from their intended path, ensuring that their actions and decisions contribute positively to their overall business performance [54]. In summary, financial availability catalyzes entrepreneurial performance among rural households by generating a strong motive rooted in the obligation to repay loans. This motive fuels their entrepreneurial drive, guides their strategic choices, and bolsters their resilience, ultimately leading to improved business outcomes [55].

### 2.2. Theoretical analysis of behavioral conditions

Research has consistently shown that economies of scale are a significant driver of entrepreneurial success, especially in the field of agriculture [56–58]. One of the key mechanisms through which financial availability promotes rural households’ entrepreneurial performance is by enabling entrepreneurial rural households to scale their business operations, thereby achieving economies of scale. When rural households have access to financing, they can invest in expanding their production capacity, purchasing additional equipment, and hiring more labor, among other things. This scaling up allows rural households to move away from small-scale, inefficient, and high-cost family-based production models and transition towards larger-scale, more efficient operations. The benefits of achieving economies of scale are manifold. Larger production volumes can lead to cost savings due to bulk purchasing of inputs, optimized resource allocation, and more efficient use of fixed costs. Furthermore, economies of scale can enhance rural households’ ability to compete in the market, as larger operations often have the capacity to invest in marketing, branding, and research and development, which can differentiate their products and attract more customers.

Meanwhile, in the mechanism of financial availability in promoting entrepreneurial performance, a crucial intermediary link is the acquisition of “legitimacy” status through the organized operation of rural households’ entrepreneurial projects. Legitimacy refers to the general recognition and assumption of whether the actions of an entity conform to expectations, appropriateness, and suitability within a socially constructed system of beliefs, norms, and values. Simply put, it represents the degree to which an organization can be accepted and recognized by society [59,60]. In the process of rural households’ entrepreneurship, the acquisition of legitimacy significantly impacts the enhancement of entrepreneurial performance. Firstly, entrepreneurial orientation, entrepreneurs’ social skills, and political connections play a vital role in obtaining legitimacy for nascent enterprises [61–63]. These factors collectively act on nascent enterprises, helping them establish trust and recognition in rural society, thus making it easier to overcome the disadvantages of being new. Secondly, the acquisition of legitimacy makes rural households’ entrepreneurial projects more easily recognized by rural communities and interpersonal relationships. In rural societies, interpersonal relationships and social networks are crucial for the survival and development of enterprises. Enterprises with legitimacy can more easily integrate into these networks, obtaining more resources and support [64]. Furthermore, enhanced legitimacy makes the brand of nascent enterprises more easily accepted by society. In the eyes of consumers and the market, enterprises with legitimacy often imply higher credibility and more reliable product quality. This helps enterprises build their brand image, enhance market competitiveness, and directly improve entrepreneurial performance [61]. Lastly, the acquisition of legitimacy enriches the transaction records between enterprises and society, reducing the risks and uncertainties associated with the lack of legitimacy. An enterprise widely accepted and recognized by society enjoys more conveniences and advantages in financing, cooperation, sales, and other aspects, thereby reducing various risks in the entrepreneurial process [65].In summary, financial availability promotes the acquisition of “legitimacy” status through the organized operation of rural households’ entrepreneurial projects, thereby enhancing entrepreneurial performance. The acquisition of legitimacy not only makes it easier for rural households to overcome nascent disadvantages and gain social recognition and support but also enhances the brand influence and market competitiveness of enterprises, reducing entrepreneurial risks and laying a solid foundation for the success of rural households’ entrepreneurship.

### 2.3. Analysis of differences in the role of formal and informal financial availability

Rural financial markets in developing countries are typically characterized by a dualistic structure. In this structure, the formal and informal finance sectors coexist and each has its own advantages [31,66]. Formal finance under formal institutional arrangements has advantages of scale and cost, while informal finance under informal institutional arrangements has information advantages that are embedded in rural networks of people [67]. This study explores the differences in their roles by analyzing the fit between the dominant characteristics of formal and informal finance and the entrepreneurial performance growth behavioral intentions and behavioral conditions of rural households.

In terms of behavioral intentions, formal finance is more motivating for entrepreneurial rural households to improve their performance. The reason is that the formal finance loan contract is based on a formal institutional arrangement of contracts, and the entrepreneurial rural households who have access to formal finance have a specific deadline for loan repayment, which is agreed upon in the contract and cannot be changed arbitrarily. In contrast, informal finance loan contracts are based on interpersonal trust in informal institutional arrangements, where the loan repayment period tends to be more ‘flexible’ and therefore provides weaker incentives.

In terms of behavioral conditions, formal finance is still more advantageous. The reason is that the expansion of rural households’ entrepreneurial scale and the realization of organized management are accompanied by a larger scale of capital demand, and formal finance based on institutional trust has the scale advantage and can satisfy a larger scale of capital demand, while informal finance based on interpersonal trust is limited by the scale of capital and the scope of the relationship network, and has a relatively limited role. This shows that the scale advantage of formal finance is a better match for the expansion of rural households’ entrepreneurial scale and the characteristics of the capital demand of organised management.

### 2.4. Regional heterogeneity based on non-financial constraints

Beyond the inherent differences between formal and informal finance, the impact of financial availability on entrepreneurial performance may be moderated by regional non-financial constraints, which could lead to potential regional heterogeneity. Rural areas in southern China typically feature denser urban networks, more vibrant commodity economies, smoother market access for both agricultural and non-agricultural products, and lower market entry barriers [68]. These regions also boast more advanced infrastructure and richer entrepreneurial support services that enhance entrepreneurial households’ ability to convert large-scale formal financial support into production expansion, brand building, and operational efficiency improvements, thereby potentially amplifying the positive effect of formal finance on performance. In contrast, rural areas in northern China are with relatively moderate marketization levels. Their development is characterized by a greater emphasis on staple crop cultivation and relatively concentrated industrial structures, which translates to a more gradual pace in the construction of diversified market channels and supporting infrastructure. Entrepreneurial support resources are also distributed in a more concentrated manner across key areas. Even with access to formal finance, the relatively steady development rhythm of local markets and the gradual improvement of supporting conditions may constrain the speed at which financial resources are converted into tangible performance gains, potentially softening the promotional role of formal finance to some extent. Thus, disparities in non-financial constraints including market access, infrastructure, and managerial capabilities between northern and southern rural areas may form the boundary condition for the impact of financial availability on entrepreneurial performance.

### 2.5. Research hypotheses

Based on the above analyses, the hypotheses of this study were formulated:

**Hypotheses1.1:** Formal financial availability positively contributes to the entrepreneurial performance of rural households.

**Hypotheses1.2:** Informal financial availability has significant effect on entrepreneurial performance of rural households.

**Hypotheses2:** Formal financial availability plays a more important role than informal financial availability.

**Hypotheses3:** There is regional heterogeneity in the effect, with a stronger effect in southern rural China.

## 3. Materials and methods

### 3.1. Data sources

This paper uses data from the ‘China Family Panel Studies (CFPS)’, which is implemented by the China Social Science Survey Centre (ISSS) of Peking University, the questionnaire covers 25 provinces (districts and municipalities) nationwide, and has been conducted every two years since 2010. Combined with the actual research, this paper takes the 2016 entrepreneurial households as the benchmark sample, and the entrepreneurial situation of the benchmark sample of households in the 2018 tracking survey as the control, to empirically study the impact of financial availability on the entrepreneurial performance of agricultural households, and the final micro-sample used in this paper consists of 554 rural entrepreneurial households after eliminating the samples of urban households, non-entrepreneurial households, and the samples of key variables that are missing.

### 3.2. Definition of variables and descriptive statistics

#### 3.2.1. Dependent variable.

This paper focuses on the impact of financial availability on the growth of entrepreneurial performance of rural households in the continuous growth stage of their entrepreneurship. Therefore, the dependent variable in the study of this paper is the growth of entrepreneurial performance of rural households. Many scholars have conducted research on measuring the level of entrepreneurial performance of rural households, which mainly involves two categories: subjective evaluation and objective evaluation. Among them, the subjective evaluation method is mainly based on the subjective satisfaction of the rural households themselves with the various results of entrepreneurial behavior as a measure, and it is common to use the Likert scale method for direct assignment or relative assignment based on the comparison between different rural households [5,69,70]. The objective evaluation category is based on the financial perspective, based on indicators such as operating income and profit generated by the rural households’ entrepreneurship [71,72]. In addition, some scholars have adopted a combination of subjective and objective evaluation indexes, and then used factor analysis and other methods to quantify the dimensionality or cluster analysis [9]. Each of the above methods has its own advantages and can reflect the performance level of rural households’ entrepreneurship to a corresponding extent, which provides rich experience for this paper to measure and evaluate the performance of rural households’ entrepreneurship. However, measuring the entrepreneurial performance of rural households through subjective evaluation methods may lead to greater subjectivity and arbitrariness in the results due to the different feelings of the rural households’ subjects. The current trend in evaluating entrepreneurial performance is to use objective indicators for evaluation [73]. In rural areas of developing countries, entrepreneurship among rural households is aimed at income growth, asset enhancement and improvement in living standards. Therefore, this paper believes that the variables reflecting the growth degree of entrepreneurial performance of rural households can be obtained through a comprehensive examination of the relevant indicators of rural households’ income, assets and living standards. In terms of specific measurement methods, this paper uses SPSS software to select relevant indicator variables, and applies factor analysis for dimensionality reduction.

#### 3.2.2. Independent variable.

In this paper, the total financial stock of the formal and informal financial sectors to rural households is set as the independent variable of the model. The data are taken from the ‘Financial Assets and Debts’ section of the CFPS questionnaire. In this paper, the aggregate principal and interest balances of loans received by rural households from banks and non-bank formal financial institutions are used as the formal financial availability variable, and the aggregate principal and interest balances of loans received by rural households from relatives, friends, and other members of the community are used as the informal financial availability variable. This approach takes into account the relevant definitions of formal and informal finance in the existing literature [31,67].

#### 3.2.3. Controlled variable.

This paper refers to the existing relevant studies on the factors influencing the entrepreneurial performance of rural households to determine the control variables to be added [70,74]. Firstly, the variables in terms of rural household characteristics are controlled based on different dimensions, controlling household characteristics such as marital status of the head of household, age, gender, and the highest level of education of family members. Second, the variables in terms of social capital of rural households are controlled. Specifically, rural household gift expenditure is selected as a proxy variable for social capital. At the same time, the number of people eating at the same stove and whether they are party members are added to control the level of social capital of rural households more comprehensively. This is because there may be a large regional bias in gift expenditures across customs. In addition, the external environment of the region where the rural household is located may also affect the level of entrepreneurial performance growth of the rural household to a greater extent. Therefore, in terms of local conditions by adding regional GDP per capita variable to control the level of local economic development. At the same time, the factor marketisation index of the region is added to control the degree of marketisation of the region where the entrepreneurial rural households are located.

#### 3.2.4. Factor analysis of dependent variables.

As the dimensions covered by the growth of entrepreneurial performance of rural households are relatively rich in connotation, this paper intends to use factor analysis to reduce the dimensionality through a comprehensive examination of the indicators related to the three dimensions of rural households’ income, assets and living standards. Firstly, the growth of entrepreneurial performance of rural households will directly bring the increase of income to rural households. On the dimension of income, this paper selects four indicators of entrepreneurial profit, profit growth, total household income, and per capita household income of rural households in CFPS survey; secondly, assets reflect the degree of wealth accumulation of entrepreneurial rural households. In the dimension of assets, this paper selects four indicators: savings deposits, total household assets, net household assets and fixed assets; in addition, the level of consumption-related expenditures of rural households’ households reflects their living standards more objectively. Therefore, this paper selects six indicators: value of consumer durables, housing expenditure, daily life expenditure, clothing expenditure, education expenditure and food expenditure. Combining the above aspects, this paper selects a total of 14 indicators in three dimensions: entrepreneurial profit of rural households (Y1), profit growth in two surveys (Y2), total household income (Y3), per capita household income (Y4), savings deposits of rural households (Y5), total household assets (Y6), household net assets (Y7), fixed assets (Y8), value of consumer durables (Y9), housing expenditures (Y10), daily life expenditures (Y10), and food expenditures (Y11). Y10), expenditure on daily living (Y11), expenditure on clothing (Y12), expenditure on education (Y13), and expenditure on food (Y14).

On this basis, this paper first needs to deal with the problem of missing data. The vast majority of the relevant indicators selected from the CFPS questionnaire have been directly given clear answers by the interviewed entrepreneurial rural households in the survey, and the responses are continuous variables with specific values. However, the measurement of the entrepreneurial performance variables is based on the factor analysis of 14 indicators, which involves many indicators, and it is difficult to avoid the problem of missing data, for example, a small number of entrepreneurial rural households did not answer all the questions in the 14 indicators when they were interviewed. The CFPS survey involved a full sample of nearly 16,000 households in both rural and urban areas, but after screening by the criteria of ‘rural households’ and ‘having started a business’, less than 600 entrepreneurial rural households remained in the survey sample. If the sampled households with more than one missing indicator were to be excluded directly, a large proportion of the survey sample would be lost. The treatment of the 14 indicator variables of the sample rural households in this paper needs to be done in order to avoid statistical bias caused by too much missing data on the one hand, and on the other hand, it also needs to prevent the problem of the empirical sample size being too small caused by the simple exclusion of the sample with missing data. Therefore, we drawn on applied research on missing interpolation methods for statistical data [75]. In this paper, rural household income is used as the main rule of association, and the nearest neighbour interpolation method is applied to interpolate some of the missing data to ensure the completeness and uniformity of the variable data. Table 1 provides the descriptive statistics of the data of the processed original variables of the factor analysis of growth of entrepreneurial performance of rural households.

**Table 1:** Descriptive statistics of raw variables for factor analysis.

Variable	Descriptions	Mean	Std. Dev.	Min	Max
Y1	entrepreneurial profit	4.057	7.907	−5	100
Y2	profit growth	−1.626	12.09	−200	95
Y3	household income	13.76	55.93	0.27	1139
Y4	income per capita	3.228	10.35	0.1	162.7
Y5	savings deposits	4.641	8.696	0	75
Y6	total assets	36.60	73.67	0	1000
Y7	net assets	33.05	72.52	−25	1000
Y8	fixed assets	18.06	55.67	0	700
Y9	durables	6.838	11.08	0	100
Y10	housing expenditures	1.320	3.068	0	42.16
Y11	daily life expenditures	1.850	5.463	0	53.12
Y12	expenditure on clothing	0.331	0.389	0	3
Y13	expenditure on education	0.654	1.007	0	10.50
Y14	expenditure on food	1.933	1.892	0	20.40

Note: (1) Negative profits from rural household start-ups indicate that rural household start-ups have incurred financial losses; (2) negative profit growth between the two surveys indicates that profits from rural household start-ups have declined from the base survey period to the next survey period; and (3) negative household net worth indicates that rural household liabilities exceed assets.

Secondly, before the formal factor analysis of the level of entrepreneurial performance of rural households, it is necessary to carry out a reliability test on the processed data of the original variables to determine the reliability of the data of the original variables. The reliability test Cronbach’s coefficient α is 0.812, which indicates that the internal consistency of the selected 14 original variables is good and the reliability is high. Meanwhile, factor analysis is designed to extract representative common factors from many variables, and the method requires the existence of a certain correlation between the original variables. In this paper, KMO (Kaiser-Meyer-Olkin) and Bartlett Test of Sphericity (Bartlett Test of Sphericity) spherical methods were used to test the correlation of the selected questions. The results of the test show that the KMO value is 0.757, indicating that there is a strong correlation between the 14 original variables selected to measure the growth of entrepreneurial performance of rural households; the p-value of the test statistic of sphericity is 0.00, indicating that the data are suitable for factor analysis.

In addition, factor analysis needs to be combined with variance decomposition to determine the number of common factors. When the number of common factors is 4, the cumulative variance contribution has reached 67.695%. The marginal effect of the first 4 common factors on the eigenvalue of the target variable is large, and they can already reflect the characteristics of the target variable to a large extent. And the marginal effect on the eigenvalue inscription of the target variable decreases rapidly from the 5th common factor, so the selection of the first 4 common factors is relatively appropriate. Meanwhile, referring to the existing literature on the use of factor analysis to measure the rural households’ variables, the cumulative variance contribution rate of the public factors reaches more than 60%, then most of the information of the target variables is reflected [76–78], which indicates that the extracted public factors can better cover the level of growth of entrepreneurial performance of the rural households. Therefore, this paper extracts the first 4 metrics of the analysed results.

Finally, it is also necessary to understand the dimensions of rural households’ entrepreneurial performance level covered by the public factors. In this paper, orthogonal rotation transformation is performed by SPSS software to get the rotated factor loadings to analysis which information about the growth level of entrepreneurial performance of rural households is mainly carried by the public factors. Meanwhile, the rotated public factor score coefficient matrix was calculated to obtain the four public factor scores. The performance growth level of each sample entrepreneurial rural households was calculated by weighting and summing the public factor scores using the percentage of variance of the rotated loadings of each factor as weights. Table 2 shows the factor loading information after rotation of the 14 variables.

**Table 2:** Factor loadings after rotation.

Public factors	Variable	Descriptions	Factor loading
F1	Y1	entrepreneurial profit	0.887
	Y2	profit growth	0.857
	Y3	household income	0.698
	Y4	income per capita	0.793
	Y5	savings deposits	0.427
	Y8	total assets	0.610
	Y9	net assets	0.692
F2	Y12	fixed assets	0.457
	Y13	durables	0.455
	Y14	housing expenditures	0.450
F3	Y6	daily life expenditures	0.692
	Y7	expenditure on clothing	0.678
F4	Y10	expenditure on education	0.714
	Y11	expenditure on food	0.465

Note: Extraction method is principal component analysis; rotation method is Kaiser standardized maximum variance method.

#### 3.2.5. Descriptive statistics.

At this point, the paper is ready with the dependent, independent, and control variables needed for the study. Table 3 shows the descriptive statistics of the values and distribution of the variables.

**Table 3:** Descriptive statistics.

Variables	Value	Mean	Std. Dev.	Min	Max
Entrepreneurial performance	Factor Analysis	1.207	0.398	−0.400	3.366
Formal Financial Availability	Actual value	2.268	9.749	0	150
Informal financial availability	Actual value	1.692	8.495	0	150
Marriage of Head of Household	0 or 1	0.906	0.292	0	1
Age of head of household	Actual value	43.64	12.73	18	80
Sex of head of household	0 or 1	0.622	0.485	0	1
Highest level of education	1 - 8	2.470	1.313	1	6
Expenditure on gifts	Actual value	0.576	0.828	0	10
Number of people	Actual value	3.863	1.915	1	15
Whether CPC member	0 or 1	0.0904	0.287	0	1
GDP per capita	Logarithm	4.914	1.922	2.746	11.51
Marketisation Index	Actual value	592	6.530	1	9.65

Note: The highest education variable is the highest level of education among the members of the farming household, with higher values indicating higher education.

### 3.3. Econometric model specification

This paper constructs an econometric regression model containing dependent, independent and control variables as follows:


$${EntrepResult}_{i}=\alpha +\beta {CreFormal}_{i}+\gamma {CreInformal}_{i}+\varphi X_{i}+\varepsilon_{i}$$

In the model, ${EntrepResult}_{i}$ represents the level of entrepreneurial performance of the rural household, α is a constant term. ${CreFormal}_{i}$ represents the formal financial availability to rural households, β is its estimated coefficient; ${CreInformal}_{i}$ represents the informal financial availability to rural households, γ is its estimated coefficient. $X_{i}$ is the control variable, φ represents the estimated coefficient of the control variable and $\varepsilon_{i}$ is the error term.

## 4. Results and discussion

### 4.1. Baseline regression

In this paper, we gradually add control variables in terms of entrepreneurial rural households’ family characteristics, social capital, and local conditions for empirical testing with Stata15.1 software. Table 4 Models 1–4 reflect the effect of explanatory and control variables on the level of entrepreneurial performance of rural households under different scenarios.

**Table 4:** Effect of financial availability on entrepreneurial performance of rural households.

	(1)	(2)	(3)	(4)
Formal Financial Availability	0.0112***	0.0106***	0.0088***	0.0077***
	(0.0022)	(0.0021)	(0.0020)	(0.0020)
Informal Financial Availability	−0.0011	−0.0019	−0.0004	−0.0000
	(0.0019)	(0.0019)	(0.0018)	(0.0018)
Marriage of Head of Household		0.0857	0.0331	0.0523
		(0.0567)	(0.0557)	(0.0549)
Age of head of household		−0.0039***	−0.0027**	−0.0027**
		(0.0013)	(0.0013)	(0.0013)
Sex of head of household		0.0048	0.0020	0.0057
		(0.0340)	(0.0325)	(0.0318)
Highest level of education		0.0469***	0.0520***	0.0480***
		(0.0142)	(0.0136)	(0.0134)
Expenditure on gifts			0.1287***	0.1254***
			(0.0189)	(0.0185)
Number of people			0.0333***	0.0319***
			(0.0086)	(0.0085)
Whether CPC member			−0.0575	−0.0344
			(0.0545)	(0.0535)
GDP per capita				0.0391***
				(0.0083)
Marketisation Index				−0.0040
				(0.0081)
constant	−0.0261	−0.0711	−0.2782***	−0.4439***
	(0.0173)	(0.0923)	(0.0939)	(0.1207)
N	554	531	531	531
R2	0.0523	0.1051	0.2060	0.2433

Note: (1) ***, **, and * denote significance levels of 1%, 5%, and 10%, respectively, and the numbers in parentheses are p-values. (2) There is a small difference in the number of model observations in the table due to missing data for a few variables.

Formal financial availability (FFA) has a positive effect on entrepreneurial performance and remains statistically significant at the 1% level across all models—consistent with Hypothesis 1.1. In the fully controlled model, FFA’s coefficient is 0.0077***: a one-standard-deviation increase in FFA (9.749 units) is associated with a 0.0077 × 9.749 ≈ 0.075-unit rise in entrepreneurial performance, equivalent to a 6.2% improvement over the sample mean (0.075/ 1.207 ≈ 0.062). This confirms FFA’s impact is not only statistically robust but also practically relevant for rural households’ post-entry growth. In contrast, the coefficient of informal financial availability (IFA) is negative but statistically insignificant across all models —thus Hypothesis 1.2 is not supported. There is a context-driven explanation. IFA’s role may center on consumption smoothing rather than investment: Rural IFA often addresses short-term liquidity gaps (e.g., unexpected household expenses or temporary operational shortages) rather than long-term performance-enhancing activities. With the above results, hypothesis 1.1 of this paper has been tested, while hypothesis 1.2 has not been tested. Hypothesis 2 is simultaneously tested.

The regression results in terms of control variables are largely consistent with the findings of the existing literature on the factors influencing entrepreneurial performance. First, the estimated coefficient of age of the household head is negative and significant. This reflects the negative relationship between age and performance, with younger rural entrepreneurial households tending to have better entrepreneurial performance. Secondly, the estimated coefficients of highest education and household size are positive and significant. This represents that human capital is important for entrepreneurial rural households to improve their business performance. In addition, the estimated coefficient of gift expenditure is significantly positive, which indicates that social relations also have an enhancing effect on the entrepreneurial performance of rural households. Finally, the estimated coefficient of regional GDP per capita is significantly positive, reflecting that the level of local economic development has an enhancing effect on the entrepreneurial performance of rural households.

### 4.2. Treatment of endogeneity

The results of the baseline regression indicate that formal financial availability promotes the entrepreneurial performance of rural households, while informal financial availability has no significant effect on the entrepreneurial performance of rural households. However, the causal identification strategy of econometric modelling is usually challenged by the endogeneity problem, which tends to bias the estimation results, leading to a huge gap between the formulation of theoretical policies and the implementation of the actual situation. Econometrics generally considers the endogeneity problem to be caused by three main factors: reverse causation, omitted variables and measurement error. First, reverse causation explains why the explanatory variables will be the core explanatory variables. The dependent variable in this paper is the entrepreneurial performance of rural households and the independent variables are the availability of formal and informal finance. If a region has a favourable business environment, the atmosphere of rural households’ entrepreneurship is strong. This situation will create a large demand for finance, and both the formal and informal financial sectors, which seek to maximize profits, will congregate in the area. This means that there is a potential for a reverse causality effect whereby rural household entrepreneurship affects both formal and informal finance. Second, although this paper has controlled for factors affecting the entrepreneurial performance of rural households as much as possible. However, there are still many observable and unobservable factors affecting the entrepreneurial performance of rural households that are not included in the econometric model and there may be an omitted variable problem. Thirdly, the CFPS database is survey data, and there is inevitably human and non-human interference, leading to the problem of measurement error in the data. Combining these three points, this paper argues that the two independent variables, formal and informal finance, are endogenous and that the results of the baseline regression may be biased. To mitigate the endogeneity problem of econometric models, it is generally necessary to find instrumental variables for the core explanatory variables. And a good instrumental variable is often difficult to find.

In this paper, the average of formal and informal financial availability is selected as the instrumental variable. The reasons are as follows: on the one hand, the mean is generated as an arithmetic average of the formal and informal finance data for each rural household in the CFPS database, which satisfies the principle of correlation of instrumental variables. On the other hand, the mean can be regarded as a smoothing indicator of the formal and informal finance data of different rural households in each region, which filters out the variability of different rural households in each region, blocks the link between the mean and other confounding factors affecting the explanatory variables, and satisfies the principle of exogeneity of the instrumental variables.

Table 5 presents the results of the instrumental variable (IV) analysis. In the first-stage regressions (models 5 and 7), the village-average formal financial availability and village-average informal financial availability—our chosen instruments—exhibit strong and significant explanatory power for individual-level financial availability. Formal validity tests support the instrument strength: The Anderson LM statistics reject the null hypothesis of under-identification, indicating the instruments are correlated with the endogenous variables. The Cragg-Donald Wald F statistics both exceed the Stock-Yogo critical value (16.38) at the 10% significance level, ruling out weak instrument concerns. In the second-stage regressions (models 6 and 8), formal financial availability exerts a positive and significant impact on entrepreneurial performance growth, while the effect of informal financial availability is positive but not statistically significant. These findings, robust to endogeneity correction, align with our theoretical expectation that formal finance—with its stability and suitability for scaling—plays a more critical role in driving post-entry performance growth among rural entrepreneurial households.

**Table 5:** Endogeneity test based on instrumental variables.

	(5) First stage	(6) Second stage	(7) First stage	(8) Second stage
Formal Financial Availability		0.0052***		
		(0.0030)		
Formal Financial Availability (village average)	1.2646***			
	(0.0651)			
Informal Financial Availability				0.0037
				(0.0045)
Informal Financial Availability (village average)			2.7623***	
			(0.2868)	
Control Variables	Added	Added	Added	Added
N	531	531	531	531
Anderson LM statistic	223.369***		80.515***	
Cragg-Donald Wald F statistic	376.844*** [16.38]		92.761*** [16.38]	

Note: ***, **, * denote significance levels of 1%, 5% and 10%, respectively; standard errors of the estimated coefficients are in parentheses for the models, and the critical value of the Stock-Yogo test at the 10% level is in middle parentheses.

To further validate the exogeneity of our instrumental variables, we conduct a placebo test by regressing base-period household income on the two instruments, while controlling for the same set of household and village-level covariates as in the main IV analysis. The results show that neither the village average of formal financial availability nor the village average of informal financial availability exerts a statistically significant impact on base-period income. This non-significant relationship confirms that our instruments do not directly influence pre-existing household economic conditions—ruling out the concern that they might operate through confounding channels unrelated to individual-level financial availability.

### 4.4. Heterogeneity discussion

To further explore the differential effects of financial availability on rural households’ entrepreneurial performance and respond to the concern about potential heterogeneous impacts across different contexts, this study conducts subgroup regressions from two dimensions: regional differences (North vs. South China) and entrepreneurial industry types (agricultural vs. non-agricultural entrepreneurship). The regression results are presented in Table 6.

**Table 6:** Heterogeneity discussion.

	(9) North	(10) South	(11) Agri	(12) Non-Agri
Formal Financial Availability	0.0066*** (0.0021)	0.0152*** (0.0053)	0.0068** (0.0028)	0.0065* (0.0033)
Informal Financial Availability	−0.0010 (0.0025)	−0.0004 (0.0028)	0.0003 (0.0018)	0.0063 (0.0063)
control variables	Added	Added	Added	Added
N	313	218	331	200
R2	0.2495	0.2834	0.1939	0.3446

Note: ***, **, and * indicate significance at the 1 per cent, 5 per cent, and 10 per cent levels, respectively, and the standard errors of the estimated coefficients are in parentheses in the model.

From the perspective of regional heterogeneity, formal financial availability exerts a significantly positive effect on entrepreneurial performance in both northern and southern rural areas, but the magnitude of the effect varies notably between the two regions. Specifically, the coefficient of formal financial availability in southern China is 0.0152, which is significantly higher than the coefficient of 0.0066 in northern China. This indicates that formal finance plays a more prominent role in boosting entrepreneurial performance in southern rural areas. A plausible explanation for this regional disparity lies in the more developed rural financial markets, better infrastructure, and more active entrepreneurial atmosphere in southern China [68], which can better complement the role of formal financial support and thus amplify its positive impact on entrepreneurial performance. In contrast, informal financial availability shows no significant correlation with entrepreneurial performance in either northern or southern regions, suggesting that the limited role of informal finance is consistent across different regional contexts. With the above results, hypothesis 3 is tested.

In terms of industry heterogeneity, the regression results for agricultural and non-agricultural entrepreneurship subgroups show that formal financial availability remains significantly positive in both subgroups, with coefficients of 0.0068 and 0.0065 respectively. The coefficients are very close in magnitude, indicating that the promoting effect of formal financial availability on entrepreneurial performance does not differ significantly between agricultural and non-agricultural entrepreneurship. For informal financial availability, its coefficients are positive but insignificant in both the agricultural and non-agricultural subgroups, which further confirms that informal finance fails to exert a significant effect on the performance growth of rural households’ entrepreneurial activities, regardless of the industry type.

Overall, the heterogeneity analysis reveals that the positive effect of formal financial availability on rural households’ entrepreneurial performance is subject to regional differences, while it is relatively stable across different entrepreneurial industries. Informal financial availability, however, does not show significant heterogeneous effects in either regional or industry dimensions, which further supports the core conclusion of this study that informal finance has a limited role in promoting the performance growth of rural households’ entrepreneurial activities in the post-startup phase.

### 4.3. Robustness testing

In this paper, the estimation results of the econometric model are robustly tested in two ways to further verify the reliability of the above regression results. Firstly, the indicators of rural households’ entrepreneurial performance were re-measured using principal component analysis and added to the econometric model for robustness test. Secondly, rural household entrepreneurial profit was selected as a proxy indicator for rural household entrepreneurial performance and added to the econometric model for robustness testing.

Models 13–16 in Table 7 show the results of the robustness tests for the first approach. Models 13 and 15 do not incorporate control variables, while models 14 and 16 incorporate all control variables. The estimation result is that formal financial availability enhances the entrepreneurial performance of rural households, while the role of informal financial availability is insignificant. This result is consistent with the previous tests and indicates that the results are robust.

**Table 7:** Robustness test: Approach I.

	(13)	(14)	(15)	(16)
Formal Financial Availability	0.0021*** (0.0041)	0.0104*** (0.0022)		
Informal Financial Availability			0.0039 (0.0067)	0.0040 (0.0081)
control variables	None	Added	None	Added
N	554	531	554	531
R2	0.0345	0.0624	0.0133	0.0551

Note: ***, **, and * indicate significance at the 1 per cent, 5 per cent, and 10 per cent levels, respectively, and the standard errors of the estimated coefficients are in parentheses in the model.

Models 17–20 in Table 8 show the results of the robustness tests for the second approach. Models 17 and 19 do not incorporate control variables, while models 18 and 20 incorporate all control variables. The regression results remain consistent with the previous ones, further indicating that the test is robust.

**Table 8:** Robustness test: Approach II.

	(17)	(18)	(19)	(20)
Formal Financial Availability	0.0125*** (0.0033)	0.0080** (0.0032)		
Informal Financial Availability			0.0034 (0.0038)	0.0005 (0.0037)
control variables	None	Added	None	Added
N	554	531	554	531
R2	0.0239	0.1143	0.0014	0.1044

Note: ***, **, and * indicate significance at the 1 per cent, 5 per cent, and 10 per cent levels, respectively, and the standard errors of the estimated coefficients are in parentheses in the model.

Notably, these robustness checks complement our IV-based identification strategy: While the IV test addresses endogeneity from reverse causality, omitted variables, and measurement error, the two robustness tests rule out biases from how we define and measure “entrepreneurial performance”. Together, they ensure our core conclusion—”formal financial availability promotes rural households’ entrepreneurial performance”—is reliable and not contingent on specific model or variable choices.

## 5. Conclusions

### 5.1. Study conclusions

In this paper, we selected rural households in the stage of entrepreneurial development as the research object, revealed the logical mechanism of the impact of financial availability on the growth of entrepreneurial performance of rural households from the aspects of behavioral willingness and behavioral conditions, and analyzed the difference between the impact of formal and informal finance. At the same time, we constructed an econometric model and conducted an empirical test using CFPS survey data. It is found that only formal financial availability has a significant effect on the growth of entrepreneurial performance of rural households at the stage of entrepreneurial development, while informal financial availability is not ineffective.

Further heterogeneity analysis based on regional and industry differences reveals two key findings. First, the positive effect of formal financial availability on entrepreneurial performance exhibits notable regional heterogeneity: the promotion effect of formal finance is significantly stronger in southern rural areas than in northern rural areas. Second, in terms of industry differences, the positive effect of formal financial availability on entrepreneurial performance remains stable across agricultural and non-agricultural entrepreneurship. For informal financial availability, no significant heterogeneous effects are identified in either regional or industry dimensions.

Combined with the relevant findings of the existing literature, the study in this paper makes useful additions to further clarify the panorama of the impact of financial availability on rural households’ entrepreneurship. Due to the reality of a two-track system of rural finance in developing countries, there are important structural differences between the role of formal and informal financial availability in influencing rural household entrepreneurship at different stages. In the ‘intention-preparation’ stage before start-up, both formal and informal finance are effective in facilitating the entrepreneurial choices of rural households through the mechanism of alleviating financial constraints. However, in the ‘development’ stage after the start-up, only formal finance can effectively promote the growth of entrepreneurial performance through the satisfaction of behavioral willingness and conditions, and this effect is subject to obvious regional heterogeneity.

### 5.2. Policy implications

It is important to note that the following recommendations are evidence-based insights derived from this study’s focus on post-entry rural entrepreneurial households. Given the complexity of rural financial systems, they should be flexibly adjusted to local contexts and combined with broader rural development policies.

On the one hand, strengthen formal finance support by targeting core rural credit access barriers. Rural areas in developing countries still face significant formal financial exclusion, and generic expansion of formal finance will not effectively support entrepreneurial growth without addressing well-documented obstacles. Policies should prioritize targeted measures to mitigate key barriers: for collateral requirements, this could involve developing substitutes suitable for rural households; for transaction costs, exploring tools like digital finance to streamline loan application and repayment processes; and for information asymmetries, building platforms that connect formal financial institutions with local entities to share reliable data on households’ entrepreneurial performance and risk profiles. Such targeted adjustments can help formal finance better match the post-entry capital needs of rural entrepreneurial households, rather than relying on one-size-fits-all expansion.

It is important to note that the following recommendations are evidence-based insights derived from this study’s focus on post-entry rural entrepreneurial households. Given the complexity of rural financial systems, they should be flexibly adjusted to local contexts and combined with broader rural development policies.

On the other hand, guide informal finance toward complementary, organized development. While this study finds informal finance has no significant effect on post-entry performance growth, it still plays a role in short-term liquidity smoothing for rural households. Policies should avoid pushing for full formalization and instead focus on supporting community-based informal financial groups that retain flexibility while reducing moral hazard through group supervision. Additionally, promoting collaboration between informal groups and formal institutions can leverage the strengths of both channels, creating a more inclusive support system for rural entrepreneurship.

On the other hand, guide informal finance toward complementary, organized development. While this study finds informal finance has no significant effect on post-entry performance growth, it still plays a role in short-term liquidity smoothing for rural households. Policies should avoid pushing for full formalization and instead focus on supporting community-based informal financial groups that retain flexibility while reducing moral hazard through group supervision. Additionally, promoting collaboration between informal groups and formal institutions can leverage the strengths of both channels, creating a more inclusive support system for rural entrepreneurship.

#### 5.3. Research limitations and future directions.

This study explores the impact of financial availability on the post-startup performance growth of rural households, but certain limitations persist, providing avenues for future research.

First, regarding the instrumental variable strategy, we have taken multiple steps to enhance its reliability—including conducting placebo tests, formal validity checks, and incorporating control variables measuring local development levels to account for potential confounding from village-level economic conditions. While these efforts help mitigate unintended interference, we acknowledge that the village-level instrumental variables may still partially reflect broader regional economic dynamics beyond the direct effect of individual financial availability, as fully capturing all unobserved regional factors remains a challenge in empirical research. Future research could adopt more granular instrumental variables and integrate richer regional covariates to further isolate the net effect of individual financial availability, thereby reducing potential interference from unobserved village-level economic dynamics.

Second, the sample of this study is intentionally restricted to ongoing entrepreneurial households, aligned with the core research theme of examining financial availability’s impact on the performance growth of rural households in the post-startup development stage. However, this selection means the study does not include failed or exiting entrepreneurs. We fully recognize that exploring the role of financial availability in entrepreneurial failure or exit is of great importance for a comprehensive understanding of the rural entrepreneurship ecosystem. Future research could dedicate specific efforts to this topic—focusing on failed or exiting entrepreneurs as the research object to analyze how financial availability and other factors influence entrepreneurial sustainability. This direction not only addresses the current limitation but also represents an innovative extension of the existing research, contributing more holistic insights into rural entrepreneurship.

Third, while this study has supplemented regional and industry heterogeneity analysis, informal finance is treated as a homogeneous construct due to data constraints from the CFPS survey. Detailed classifications regarding the purpose, scale, or timing of informal financial use are unavailable, which may obscure meaningful differences in its role. Future research could adopt micro-level survey data with refined informal finance classification to explore the heterogeneous effects of different types of informal finance on rural entrepreneurial outcomes, providing more targeted policy implications for the standardized development of informal finance.
